# Iliopsoas Hematoma Progression to Abscess in the Setting of Diabetic Ketoacidosis

**DOI:** 10.7759/cureus.42993

**Published:** 2023-08-05

**Authors:** William H Arata, Kunal Aggarwal, Rachel Nelson, Kosuke Iwaki

**Affiliations:** 1 Internal Medicine, St. George’s University, St. George's, GRD; 2 Internal Medicine, Elmhurst Hospital Center, New York City, USA; 3 Medical Education, St. George's University, St. George's, GRD; 4 Physical Medicine and Rehabilitation, Elmhurst Hospital Center, New York City, USA; 5 Internal Medicine, St. George's University, St. George's, GRD

**Keywords:** methicillin-sensitive staphylococcus aureus, diabetic ketoacidosis, hematoma, psoas abscess, iliopsoas hematoma

## Abstract

Iliopsoas hematomas (IPH) are defined as a spontaneous or traumatic retroperitoneal collection of blood involving the iliopsoas muscle. In some cases, intramuscular hematomas can progress to abscesses and put the patient at risk for further complications. Our objectives are: to describe the etiology of intramuscular hematoma and psoas abscess, to describe the clinical signs and treatment of intramuscular hematoma and psoas abscess, and to analyze the association between uncontrolled diabetes mellitus and psoas abscess progression, which we achieve through retrospective case analysis and associated literature review on symptom constellation. We present the case of a 40-year-old male patient with a history of diabetes mellitus and alcohol abuse who presented with three days of increasing back and left lower extremity pain, confusion, auditory hallucinations, and fever found to be in diabetic ketoacidosis. Six days prior, the patient presented to the Emergency Department (ED) after being struck by a motor vehicle while ambulating found to have bruising, weakness in his lower extremities, and an L2 vertebrae fracture found on CT. During the presentation, the patient was found to have decreased muscle strength, leukocytosis with elevated lactate, and CT findings suggestive of a left psoas abscess drained by interventional radiology. Vancomycin and Cefepime were used as an empiric antibiotic regimen. The culture of the wound was then found to grow Methicillin-susceptible Staphylococcus aureus (MSSA) bacteria and antibiotics were then adjusted to Vancomycin and Cefazolin.

During the patient’s hospital stay, he developed two more abscesses on his bilateral psoas muscles, which were promptly percutaneously drained by interventional radiology. This case describes an uncommon progression of an Iliopsoas hematoma to a psoas abscess, likely due to his immunocompromised status secondary to his uncontrolled diabetes mellitus. Uncontrolled diabetes mellitus has been shown in various studies to be an independent risk factor of intramuscular hematoma progress to psoas abscess. We suggest that patients displaying fever, chills, flank pain, limited hip movement, and indications of uncontrolled diabetes should be approached with a high degree of suspicion for a psoas abscess.

## Introduction

Iliopsoas hematomas (IPH) are defined as a spontaneous or traumatic retroperitoneal collection of blood involving the iliopsoas muscle [1]. In some cases, intramuscular hematomas can progress to abscesses and put the patient at risk for further complications [2]. IPHs are a phenomenon that has been well-documented in various demographics [2]. Several predisposing or contributing factors have been described, and the most frequent ones include minor trauma, increased abdominal pressure, anticoagulation medications, hypertension, and iatrogenic causes [3]. However, instances of such hematomas becoming abscesses have not been well documented. The occurrence of Iliopsoas abscess is relatively infrequent, with a yearly global incidence of 12 cases. However, this incidence is on the rise, mainly attributable to advancements in diagnostic imaging techniques [4]. This paper presents a case of a 40-year-old male patient with a history of diabetes mellitus and alcohol use who presented with bilateral psoas abscesses after treatment for a motor vehicle accident where he had a nondisplaced L2 vertebral body fracture.

## Case presentation

**Figure 1:**
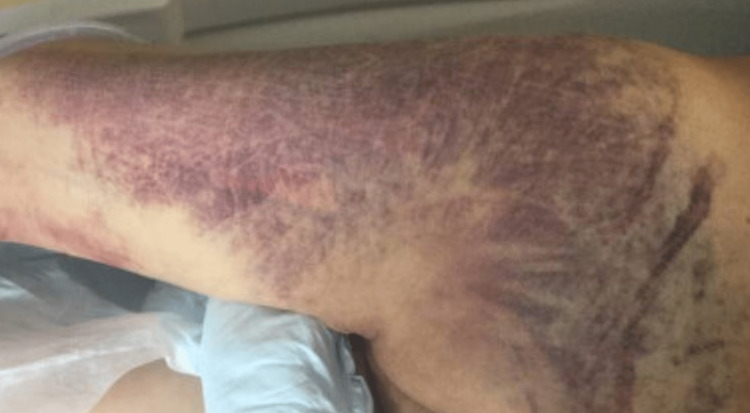
Subacute bruising of the left lower extremity

The patient is a 40-year-old male with a history of diabetes mellitus and ethanol use who was struck by a motor vehicle while ambulating in the street. The patient presented several days after the accident with complaints of worsening back pain and difficulties on ambulation. A physical exam at the time of presentation to the Emergency Department (ED) revealed exquisite midline and sacral spinal tenderness with no significant paraspinal tenderness. The patient had subacute bruising throughout his lower extremities, torso, back, and face, with bilateral bruising over his infraorbital region (Figure 1). The patient had no saddle anesthesia or lower extremity numbness or tingling. The patient did exhibit weakness in both extremities secondary to pain; however, the range of motion was intact.

**Figure 2:**
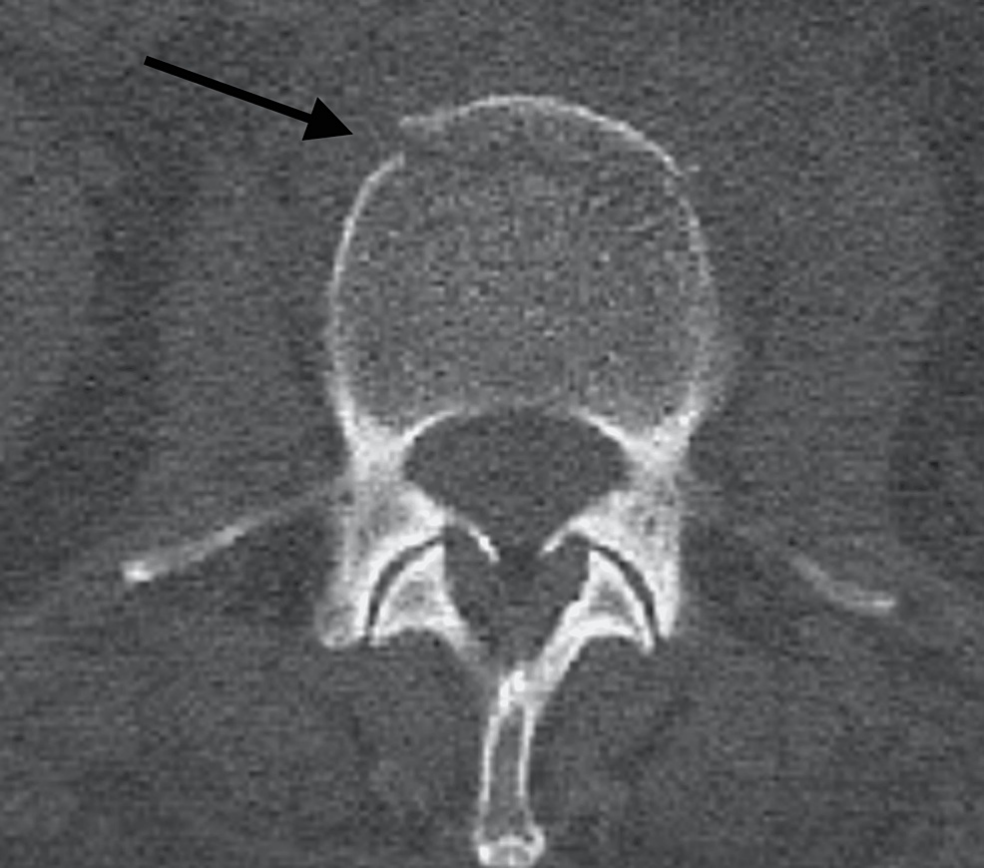
CT scan with axial view showing a nondisplaced L2 vertebral body fracture

CT spine showed a nondisplaced L2 vertebral body fracture (Figure 2). No acute surgical intervention was indicated as per the neurosurgical team, and the patient could be managed outpatient with a Thoracic-Lumbar-Sacral Orthosis (TLSO) brace and pain control with NSAIDs, Robaxin, and Gabapentin. Neurosurgery also recommended that the patient obtain MRI L-spine without contrast.

Six days after the initial presentation, the patient presented to the ED again with increasing back and left lower extremity pain, confusion, auditory hallucinations, and fever for three days. He was tachycardic to 109 bpm and febrile to 101-102F (38.8C). Neuromuscular examination demonstrated 2/5 strength in bilateral lower extremities. He was unable to perform hip flexion and leg elevation. Sensation was grossly intact throughout both lower extremities. He had a white blood cell count of 24,000/mcL with a lactate of 2.8 mmol/L. The patient was also found to be in diabetic ketoacidosis with a blood glucose of 588 mg/dL, an anion gap of 26, and a pH of 7.35. Subsequent imaging showed L2 paraspinal soft tissue mass with multilocular low attenuation changes in the left psoas muscle. At that time, there was concern for an infected hematoma or an intramuscular abscess.

**Figure 3:**
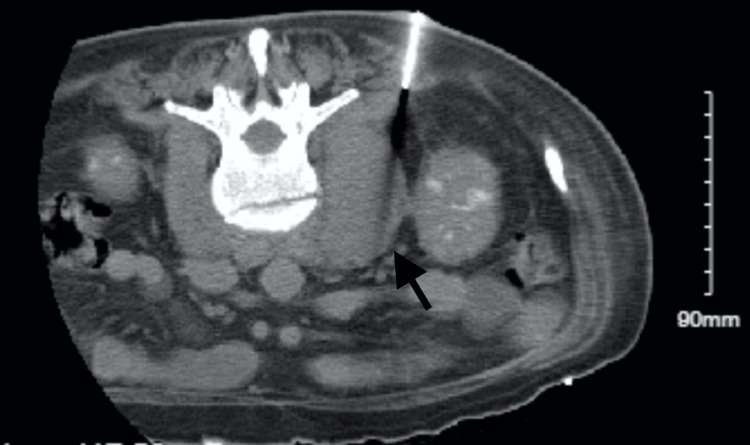
CT scan with axial view showing percutaneous drainage of left psoas abscess

He was immediately started on intravenous antibiotics, receiving two doses of intravenous vancomycin and cefepime while in the ED. He also received 2 liters of normal saline and 20meQ of potassium chloride, and he was started on an insulin drip, after which he was transitioned to insulin glargine once the anion gap was closed. Due to concern about ethanol withdrawal, the patient was given 15 mg of diazepam and 100 mg of chlordiazepoxide protocol. Interventional radiology was consulted for the drainage of the fluid collection (Figure 3). A total of 20 mL of purulent material, positive for Methicillin-sensitive Staph Aureus (MSSA) bacteria, was percutaneously drained from the left psoas muscle by IR, after which a JP drain was placed. After the procedure, he was started on an antibiotic regimen of intravenous vancomycin and cefazolin according to his wound culture speciation.

**Figure 4:**
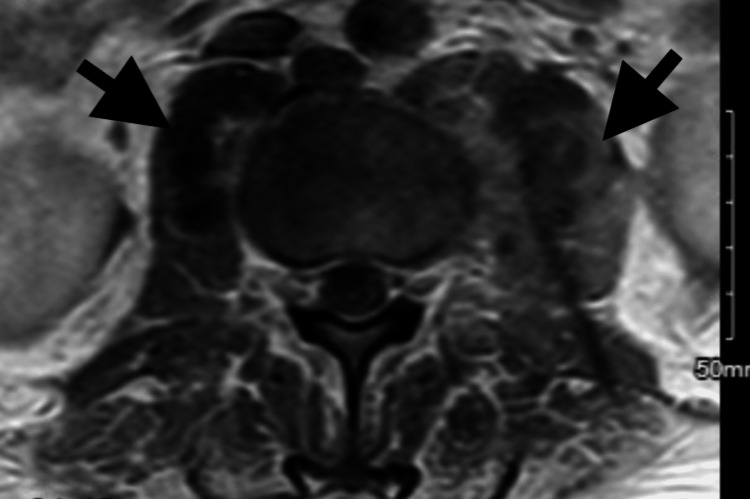
MRI with axial view showing bilateral psoas abscesses

He was transferred to the medical intensive care unit for further management. Due to concern for urinary retention, a Foley catheter was placed. An MRI was obtained as requested by the neurosurgical team, which showed bilateral psoas abscesses, an L2 fracture with edema throughout the vertebral body, and an L5-S1 disc protrusion approaching the S1 roots (Figure 4). The neurosurgical team affirmed that no surgical intervention was necessary; furthermore, they had low suspicion for urinary retention as he could spontaneously void status post Foley removal.

**Figure 5:**
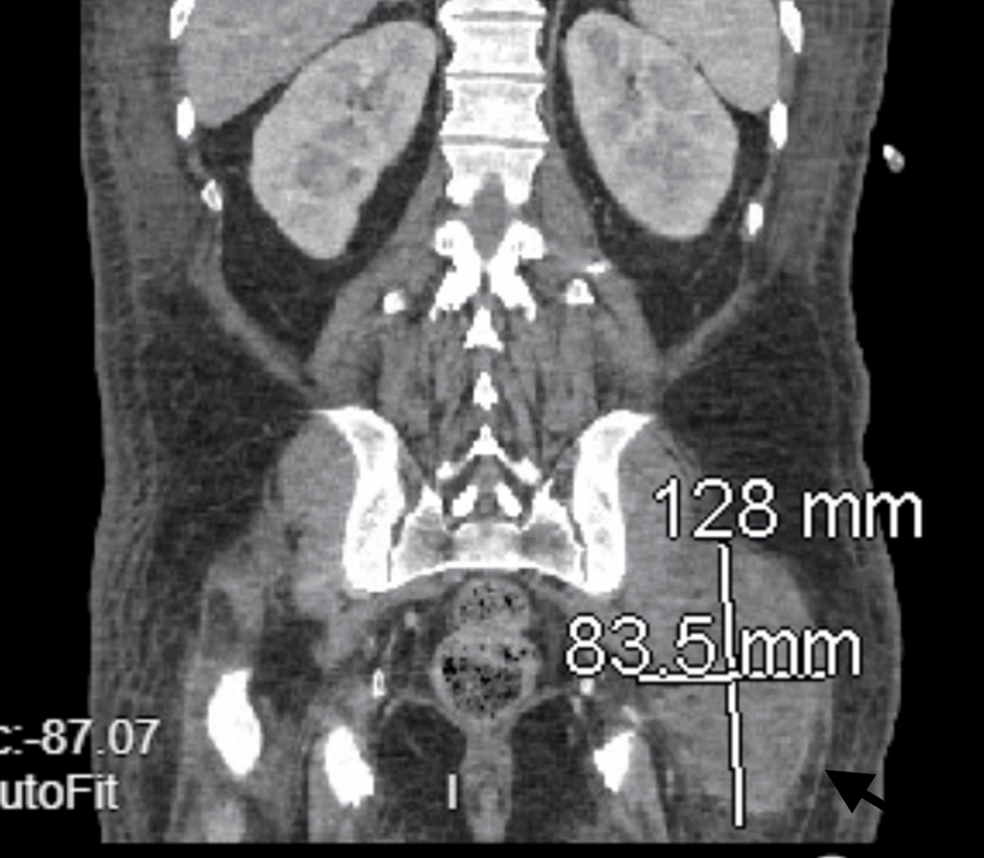
CT scan with coronal view showing new fluid collection with gas in the left gluteal region

The patient was subsequently transferred to the medicine step-down unit for the management of MSSA bacteremia, diabetes, and ethanol withdrawal. Despite clinical improvements, the presence of gram-positive blood cultures, persistent fevers, consistently elevated white blood cell count, and new findings on the repeat CT scan raised concerns regarding inadequate source control prompting another CT scan. Another CT scan revealed a new fluid collection with gas in the left gluteal region (Figure 5). Interventional radiology once again performed percutaneous drainage of the left gluteal (20 ccs of serosanguineous fluid) and right psoas (4 ccs of serosanguinous fluid) collections, with JP drains inserted. Fluid cultures yielded negative results. The patient reported symptom improvement in his left leg evidenced by 4/5 lower extremity strength testing.

The patient was then transferred to the general medicine floors, receiving active physical and occupational therapy. The neurosurgery team cleared him for weight-bearing activities. While remaining clinically stable, his pain was effectively managed with acetaminophen (650 mg every 6 hours) and daily lidocaine patch. However, he continued to experience a sensation of heaviness in his left lower extremity, particularly exacerbated by minor movements and weight-bearing. On examination, he demonstrated 3/5 strength in the right lower extremity, with a full range of motion for hip flexion and leg elevation.

**Figure 6:**
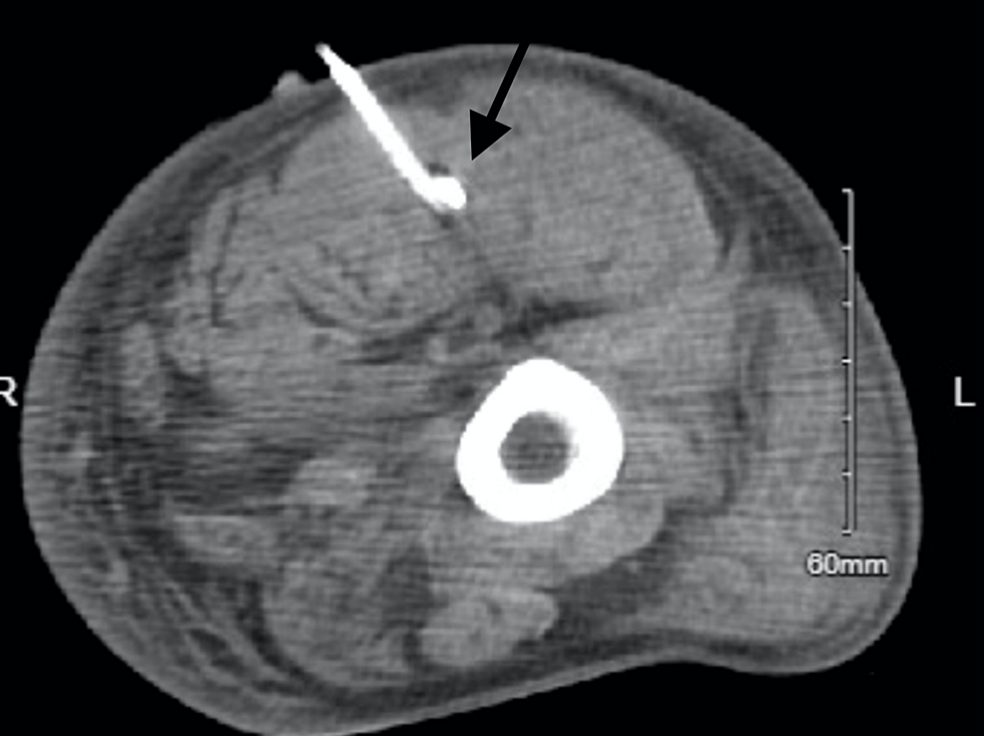
CT scan with axial view showing percutaneous drainage of a 20cm x 4.5cm x 4cm posterior thigh fluid accumulation

However, in the left lower extremity, he displayed ⅖ strength and an inability to flex the hip or elevate the leg. Furthermore, a tense loculation was identified upon palpation of the posterior thigh. Subsequent imaging disclosed a large, peripherally enhancing fluid collection measuring 20cm x 4.5cm x 4cm in the posterior thigh. Interventional radiology was once again consulted, performed percutaneous drainage of serosanguineous fluid from the posterior thigh, and placed an additional JP drain (Figure 6). Fluid cultures yielded negative results. The patient reported improvement in symptoms of the left leg. After 48 hours of no output, all four drains were removed almost one week after the procedure. The vancomycin was discontinued at this time, and the patient's intravenous cefazolin therapy was continued during his ten-week hospital stay, eventually leading to his subsequent discharge with occasional mild discomfort in the lower back and overall complete alleviation of his symptoms.

## Discussion

IPH formation is rare, with a 0.1% to 0.6% occurrence. Bilateral psoas muscle hematomas may be rarer than unilateral hematomas [5]. Risk factors for developing IPH are anticoagulation therapy, the elderly, and hemodialysis [6]. Our patient lacked any of these risk factors, which further mystified the diagnosis prior to the IPH progression to an abscess.

Trauma is a common cause of IPH formation [1], and the patient’s hematoma was likely formed during the initial car accident due to anterior non-penetrating trauma to the iliopsoas. The occurrence of incidental diagnosis is common, and the clinical presentation varies based on the severity of the hemorrhage. The clinical symptoms generally present with groin or thigh pain, muscle dysfunction, numbness or paresthesia of the unilateral lower extremity, and occasionally nerve palsy with the femoral nerve most commonly affected [7]. There were no signs of nerve palsy on our patient’s first presentation to the ED. On his return visit, he did present with decreased muscle strength in his lower extremity, likely secondary to his hematoma formation that then progressed to an abscess.

Iliopsoas abscess is an uncommon disorder caused by pus collection in the iliopsoas muscle. Primary psoas abscesses are most frequently due to infection with a single organism [8]. The most common bacterial cause is Staphylococcus aureus (S. aureus), which was identified as the infecting pathogen in our patient. In one study, S. aureus was implicated in 88 percent of cases, followed by streptococci and Escherichia coli at 4.9 and 2.8 percent [9]. The most common predisposing factors for primary abscesses are intravenous drug use and diabetes mellitus, and our case supports this finding [10]. Secondary causes may be monomicrobial or polymicrobial [11]. The infection typically arises from an adjacent structure such as the gastrointestinal tract, genitourinary system, or spine [12].

This case demonstrates uncontrolled diabetes mellitus as a risk factor for abscess formation as our patient presented to the ED with fevers and imaging suggestive of a left psoas abscess at the same time, he also presented with diabetic ketoacidosis.

The typical presentation of a psoas abscess is fever, chills, lower back or flank pain, and limitation of hip movement, with leukocytosis as the most common laboratory finding [13]. However, only one of two patients in this study presented with the classical triad of fever, flank pain, and limited hip movement. Our patient presented with the classic trio; however, due to the lack of definitive signs and symptoms in most cases, a high degree of suspicion is essential for the early diagnosis of psoas abscess.

The management approach for psoas abscess involves initiating appropriate antibiotic therapy and drainage procedures [14]. Percutaneous drainage is a good initial treatment for abscess drainage. In one study, all 29 patients with psoas abscess who underwent percutaneous intervention achieved a 100% success rate, with no reported cases of recurrence [15]. Surgical drainage may be warranted if there is a percutaneous failure.

Directed microbial therapy is generally preferable to empiric treatment. For circumstances in which prompt microbial diagnosis is not feasible, empiric antibiotic therapy should include activity against S. aureus (including activity against methicillin-resistant S. aureus in regions where prevalence is substantial) and enteric organisms (both aerobic and anaerobic enteric flora) [14]. Our patient was started on empiric therapy of vancomycin (for Methicillin-resistant S. aureus (MRSA) coverage) and cefepime (for broad gram-positive and negative coverage) and then was transitioned to vancomycin and cefazolin once the wound culture grew MSSA.

In a separate study involving 25 patients diagnosed with pyogenic psoas abscess, 65% of them had a prior history of diabetes mellitus, emphasizing the association of diabetes as a risk factor for developing diabetes mellitus [14]. Additionally, that study reported a high mortality rate of 44%, further emphasizing the importance of a high index of suspicion to enable early diagnosis of acute pyogenic iliopsoas abscess, particularly for older diabetic patients with fever, pain in the abdomen or flank, limp or flexion of the ipsilateral hip [14].

## Conclusions

This case describes an uncommon progression of an Iliopsoas hematoma to a psoas abscess, likely due to his immunocompromised status secondary to his uncontrolled diabetes mellitus. Uncontrolled diabetes mellitus has been shown in various studies to be an independent risk factor of intramuscular hematoma progress to psoas abscess. We suggest that patients displaying fever, chills, flank pain, limited hip movement, and indications of uncontrolled diabetes should be approached with a high degree of suspicion for a psoas abscess.
